# Gene and cell therapies in China: booming landscape under dual-track regulation

**DOI:** 10.1186/s13045-022-01354-9

**Published:** 2022-10-05

**Authors:** Chen Yin, Jianchao Gao, Guanqiao Li, Hongxi Hu, Liyun Zhou, Shuang Lu, Xiaoyuan Chen

**Affiliations:** 1 Tsinghua Clinical Research Institute, School of Medicine, Tsinghua University, Beijing, China; 2 National Center for Drug Evaluation, National Medical Products Administration, Beijing, China; 3 Vanke School of Public Health, Tsinghua University, Beijing, China; 4 Pharmcube (Beijing) Co., Ltd., Beijing, China; 5 Office of Clinical Trial Institute, Beijing Tsinghua Changgung Hospital, Beijing, China

**Keywords:** GCT, Clinical development, Dual-track regulation

## Abstract

The booming of gene and cell therapy (GCT) worldwide in recent years has been observed, especially in the field of cancers. In order to provide the comprehensive GCT landscape in China with a focus on differential development pathways under the current dual-track regulation mode, we analyzed 953 clinical trials initiated by March 2021 including Investigational New Drugs (IND) registered trials and investigator-initiated trials (IITs). We classified GCT products into three categories and analyzed the clinical development by phases and regulation tracks, disease areas, indications, and targets. We found that CAR-T therapies from ex vivo category and stem and somatic cells from non-gene category are two most studied therapy types and GCT mostly focused on cancers. The number of IITs far exceeded IND-registered trials except for in vivo category. After 2017, when the cell therapy guideline issued, products of all categories boomed, especially the ex vivo categories. These data showed that current dual regulation tracks in China complemented each other and together facilitated the GCT development, especially after 2017. More consistent technical standards and risk-based regulation will help bring more GCT products to patients.

**Supplementary Information:**

The online version contains supplementary material available at 10.1186/s13045-022-01354-9.

**To the Editor**

The world has witnessed the booming of gene and cell therapy (GCT) in these years, and GCT has been proved a new modality for treating incurable diseases like cancer. China ranked second only after the USA in cancer cell therapies pipeline numbers [1, 2]. However, there is a paucity of analysis of GCT pipelines with differential development pathways under the current dual-track regulation mode. In China, GCT agents can enter the “drug” track (i.e., Investigational New Drugs, IND), where their clinical trials are registered at the Center for Drug Evaluation (CDE), or the “medical technologies” track supervised by National Health Commission (NHC), where they typically initiate investigator-initiated trials (IIT) at individual hospitals. The latter can be transitioned to the “drug” track after IND submission, for the purpose of broader use. Here, we provided the latest comprehensive GCT landscape in China with a focus on these two tracks.

## Diverse types of GCT products

**Fig. 1:**
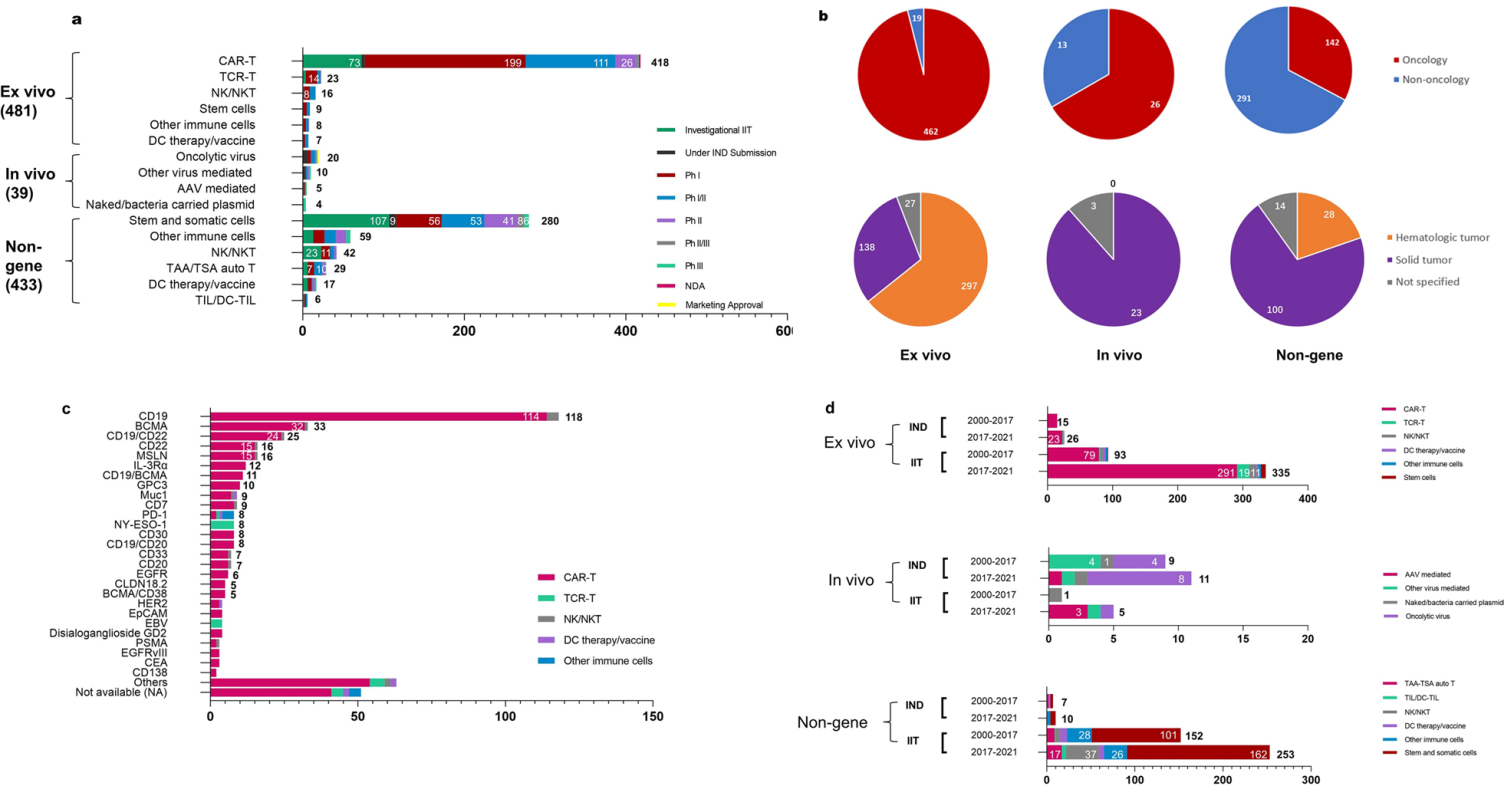
The trend of GCT clinical development by different categories in China. **a** GCT products of three categories across various therapy types by different development phases until March 2021. **b** Disease areas and indications for GCT therapies. Disease areas (oncology or non-oncology) of GCT pipelines of all three categories and general tumor types (hematologic or solid tumors) of indications of pipelines targeting oncology areas. More specific tumor types can be found in Additional file 1: Tab S1 and S2. **c** Target distribution of ex vivo therapies aiming at cancers. **d** Comparison of three categories of GCT agents undergoing IND trials and IITs, the clinical development of which initiated during 2000–2017 and 2017–2021 (including 2017 and until March 2021). The agent undergoing IND means it has registered at least one clinical trial in CDE Registration and Information Disclosure Platform, and the agent undergoing IITs means it only conducted IITs. We chose 2017 as a dividing time, as the “cell therapy guideline” issued in 2017

After near 30 years of development, the dual-track regulation mode of GCT in China has become clear since National Medical Products Administration (NMPA)’s issuance of “cell therapy guideline” in 2017 [3] and NHC’s issuance of “somatic cell therapy administration (draft)” in 2019 [4]. We classified GCT products into three categories based on the gene modification approaches (Fig. 1a) and included the agents conducting IND trials and/or IIT trials. Ex vivo categories consisted half (50.5%) of the pipelines, among which about half were under Phase I. CAR-T therapies (86.9%) dominated the ex vivo category, followed by TCR-T and CAR-NK/NKT therapies. Thanks to technology advances, a considerable proportion of CAR-T therapies stepped into late development stage, including two achieving marketing approval in 2021. Furthermore, a number of TCR-T therapies and CAR-NK therapies were tested in IITs as well as proceeding into IND-registered clinical trials since last year.

In vivo category had 39 agents, including 20 oncolytic virus products. AAV-mediated in vivo gene therapies developed rapidly in recent years in abroad [5], and a few pipelines also went into clinical stage in China. In non-gene categories, non-gene modified stem and somatic cell therapies (64.7%) dominated the pipeline, while the rest were immune cells. Among them, TAA/TSA-targeting T cells, DC and TIL therapies are all personalized adoptive cell therapies. Combination of new techniques including next-generation sequencing techniques make them promising weapons fighting against cancers. Two TIL products already received IND approval by CDE, implying a boom.

## GCT mainly focused on cancers

The majority of GCT agents targeted cancer as major indications, with the proportion of 96% (462), 67% (23), and 33% (142) in the above three categories, respectively (Fig. 1b). Hematologic tumors dominated the ex vivo category, while solid tumors took more percentages in the other two. CAR-T products have made breakthroughs in hematologic tumors with excellent efficacy. Currently, seven CAR-T products targeting either CD19 or BCMA have been approved worldwide. Unsurprisingly, these two targets were most studied in China (Fig. 1c).

The heterogeneity and tumor micro-environment of solid tumors made CAR-T cell therapies less effective. Despite this, enormous attempts were tested in solid tumors, including equipping NK cells with CAR molecule, finding new targets for CAR-T and TCR-T cells (e.g., anti-GPC3 CAR-T, anti-NY-ESO-1 TCR-T), arming CAR-T cells with cytokines [6, 7].

## Differential development paths for the three GCT categories

The dual-track regulation mode of GCT in China enables GCT to initiate clinical development by IND-registered trials or IITs. IIT trials are more flexible and can provide valuable early human data. First starting an IIT and then submitting an IND to CDE as a drug is the frequently chosen development path for lots of GCT products in China. The 2017’s “cell therapy guideline” issued by NMPA [3] with more clarified technical standards and the marketing approval of CAR-T therapies abroad in 2017 together stimulated the overall pipelines’ development. The technical standards for IIT trials also became strict and clarified recently [8], more and more equivalent to that of IND trials. Before 2017, less risky cell therapies like non-gene edited MSC therapies conducting IITs took more proportion. After 2017, both IND-registered and IIT trials boomed, especially the trials testing ex vivo categories (Fig. 1d). In vivo category displayed different trend, given that it was defined as drug since early years. The developer distribution also showed similar patterns (Additional file 1: Fig. S1).

## Outlook

We can see that current dual regulation tracks in China complemented each other and together facilitated the GCT development, especially after 2017. The regulation mode of China is different from the US mode. In the USA, GCT should apply for IND or Investigator-IND, both requiring IND application to FDA. However, regulation in China and the USA both strive for the consistent and strict standards which are the basis for steady development of GCT. Also, in the USA the biologics are regulated under section 351 and section 361 of the Public Health Services Act, and products regulated under section 361 (with relatively lower risks) need not to apply for Biologics License Application (BLA). Recently, an FDA expert’s mention of considering an intermediate regulatory pathway for some products regulated under section 361 [9] also revealed the future trend. In the future, more risk-based and stratified regulation of GCT will nurture innovation while managing risks.

IIT can provide more flexibility during R&D and IND-registered trials are more standardized. How to better connect these two pathways and keep their advantages remains a challenge. As we mentioned above, consistent technical standards and risk-based approach is the key to the GCT regulation. Thus, we suggest further issuance of consistent technical guidelines and stratified regulation based on risk for GCT clinical trials. Strengthened ethnic review is also vital for subject protection. Specific expert consensus and guidelines for GCT ethnic review were published recently in China [10, 11], and we expect more attention to be attached to patient protection. Besides regulation, greater encouragement on medical need driven R&D, and more efforts in establishing manufacturing infrastructures will help bring more novel GCT products from bench to patient in China.

## Supplementary Information

**Additional file 1:** Supplementary Methods, Table 1–2 and Figure 1.

## Abbreviations

GCT: Gene and cell therapy
IND: Investigational New Drug
IIT: Investigator-initiated trial
CDE: Center for Drug Evaluation
NHC: National Health Commission
NMPA: National Medical Products Administration
CAR: Chimeric antigen receptor
TCR: T cell receptor
NK: Natural killer
NKT: Natural killer T cell
DC: Dendritic cell
AAV: Adeno-associated virus
TAA: Tumor-associated antigen
TSA: Tumor-specific antigen
TIL: Tumor-infiltrating lymphocyte
MSC: Mesenchymal stem cell
BLA: Biologics License Application

## Declarations

### Availability of data and materials

The datasets generated and analyzed during the current study are based on Pharmcube database. The datasets used and analyzed during the current study are available from the corresponding author on reasonable request.
