# Supplementary material for: Gene and cell therapies in China: booming landscape under dual-track regulation
Source: J Hematol Oncol. 2022 Oct 5;15:139. doi: 10.1186/s13045-022-01354-9 (PMC9535931; doi:10.1186/s13045-022-01354-9)

**Supplementary Methods**

1. Data Sources

GCT pipeline clinical trial data were retrieved from Pharmcube database (one of the most authoritative platforms of drug information in China), curated from over 50 sources, including Chinese NMPA’s Registration and Information Disclosure Platform for Drug Clinical Studies, Chinese Clinical Trial Register (ChiCTR), ClinicalTrials.gov clinical trial registries, scientific conferences, company press releases, published reports, investor presentations, and other sources. The clinical trials conducted or registered in China until Mar 31th, 2021 were retrieved and analyzed.

1. Eligibility of pipelines

We adopted the GCT trials based on three categories. Each pipeline may have multiple trials and indications and we chose most advanced trial and indication. mRNA/siRNA/ASO therapies were excluded because they are chemically synthesized. Therapeutic vaccines using antigen loaded or vector transfected DCs were eligible while therapeutic vaccines with the format of peptides and proteins are excluded. Few pipelines which is hard to identify the MOA or treatment modality from publicly available information are excluded. Pipelines which are in pre-clinic period in China or publicly announced termination of development are excluded. Data were manually verified and further categorized by Tsinghua Clinical Research Institute (TCRI) and Pharmcube with parameters of therapy category, further therapy type, development stage in China, indications and target, developers and registration type. The total of 953 drug candidates included 64 therapies which entered clinical phases but the development seems no longer active; however, the presence or not of these agents does not affect the trends discussed in the article.

1. Classification and analysis of pipelines

We classify GCT products into three categories-namely, ex vivo gene therapy (gene modification of cell product occurred outside of body and the cell product delivered back into body, referred as “ex vivo category”), *in vivo* gene therapy (gene modified product delivered directly into body, referred as “in vivo category”) and non-gene introduced cell therapy (cell product without gene modification, referred as “non-gene category”). The ex vivo gene category was classified as “gene therapy”, instead of “cell therapy”, due to the higher risk compared with non-gene introduced cell therapy.

Furthermore, Ex vivo categories were sub-grouped into six types: 1) CAR-T therapy, 2) TCR-T therapy, 3) NK/NKT therapy, 4) DC therapy/vaccine therapy, 5) Other immune cells (like gene-modified CD8 T cell therapy), 6) Stem cells. In vivo categories were sub-grouped into 4 types: 1) AAV mediated therapy, 2) Other virus mediated therapy (like AdV mediated therapy), 3) Naked/bacteria carried plasmid mediated therapy, 4) Oncolytic virus therapy. Non-gene categories were sub-grouped into 5 types: 1) TAA/TSA targeted auto T cell therapy, 2) TIL/DC-TIL therapy, 3) NK/NKT therapy, 4) Other immune cell therapy (like CIK therapy, γδT therapy), 5) Stem and other somatic cell therapy (like MSC therapy). The therapy categories and types were manually verified according to publicly disclosed information. Clinical trial type included the IND-registered trials (the therapy has registered at least one clinical trial in CDE Registration and Information Disclosure Platform for Drug Clinical Studies) and IIT trials (the therapy has never registered in the above CDE platform and may only conducted NCT and/or ChiCTR platform registered trials). Developers were divided into two types: Academia and Industry. There are few pipelines developed by the cooperation of Academia and industry, and these were integrated into Academia type.

**Supplementary Table 1**. Disease indication details for GCT pipelines of three categories.

| **Category** | **Oncology/non-oncology** | **Indications** | **Pipeline No.** |
| --- | --- | --- | --- |
| ***Ex vivo*** | Oncology | Oncology | 462 |
|  | Non-oncology | Hematology | 8 |
|  |  | Infectious Diseases | 5 |
|  |  | Rheumatology | 4 |
|  |  | Neurology | 2 |
| ***In vivo*** | Oncology | Oncology | 26 |
|  | Non-oncology | Cardio-Cerebrovascular | 4 |
|  |  | Neurology | 3 |
|  |  | Ophthalmology & Otorhinolaryngology | 3 |
|  |  | Hematology | 2 |
|  |  | Endocinology | 1 |
| **Non-gene** | Oncology | Oncology | 142 |
|  | Non-oncology | Infectious Diseases | 41 |
|  |  | Cardio-Cerebrovascular | 29 |
|  |  | Musculoskeletal | 28 |
|  |  | Neurology | 24 |
|  |  | Gastroenterology | 23 |
|  |  | Gynecology | 22 |
|  |  | Ophthalmology & Otorhinolaryngology | 22 |
|  |  | Pulmonary | 20 |
|  |  | Dermatology | 20 |
|  |  | Endocinology | 14 |
|  |  | Rheumatology | 12 |
|  |  | Genitourinary | 11 |
|  |  | Transplantation | 10 |
|  |  | Others | 8 |
|  |  | Hematology | 7 |

**Supplementary Table 2**. Details of solid tumor types of three GCT categories.

| **Category** | **Indications** | **Pipeline No.** |
| --- | --- | --- |
| ***Ex vivo*** | Unspecified cancer types | 33 |
|  | Liver cancer | 18 |
|  | Brain and CNS cancer | 17 |
|  | Multiple solid tumors | 11 |
|  | Ovarian cancer | 9 |
|  | Gastric/esophagus cancer | 7 |
|  | Colorectal cancer | 6 |
|  | Pancreatic cancer | 6 |
|  | Nasopharynx cancer | 5 |
|  | Lung cancer | 4 |
|  | Prostate cancer | 3 |
|  | Kidney cancer | 3 |
|  | NSCLC | 3 |
|  | Bladder cancer | 2 |
|  | Sarcomas | 2 |
|  | Breast cancer | 2 |
|  | Head and neck cancers | 2 |
|  | Digestive tract cancers | 2 |
|  | Peritoneal carcinoma | 1 |
|  | Melanoma | 1 |
|  | Mesothelioma | 1 |
|  | **Total** | **138** |
| ***In vivo*** | Multiple solid tumors | 5 |
|  | Unspecified cancer types | 5 |
|  | Liver cancer | 3 |
|  | Nasopharynx cancer | 2 |
|  | Melanoma | 2 |
|  | Head and neck cancers | 2 |
|  | Bladder cancer | 1 |
|  | Colorectal cancer | 1 |
|  | Sarcomas | 1 |
|  | Brain and CNS cancer | 1 |
|  | **Total** | **23** |
| **Non-gene** | Liver cancer | 20 |
|  | Multiple solid tumors | 11 |
|  | Unspecified cancer types | 11 |
|  | Gastric/esophagus cancer | 11 |
|  | NSCLC | 9 |
|  | Lung cancer | 7 |
|  | Colorectal cancer | 7 |
|  | Ovarian cancer | 3 |
|  | Kidney cancer | 3 |
|  | Pancreatic cancer | 3 |
|  | Nasopharynx cancer | 2 |
|  | Melanoma | 2 |
|  | Prostate cancer | 2 |
|  | Sarcomas | 2 |
|  | Bladder cancer | 1 |
|  | Biliary tract cancer | 1 |
|  | Cervical cancer | 1 |
|  | Thyroid cancer | 1 |
|  | Breast cancer | 1 |
|  | Brain and CNS cancer | 1 |
|  | Adenoid cystic carcinoma | 1 |
|  | **Total** | **100** |

**Supplementary Fig 1**. Overview of three categories of GCT by developers and therapy types.


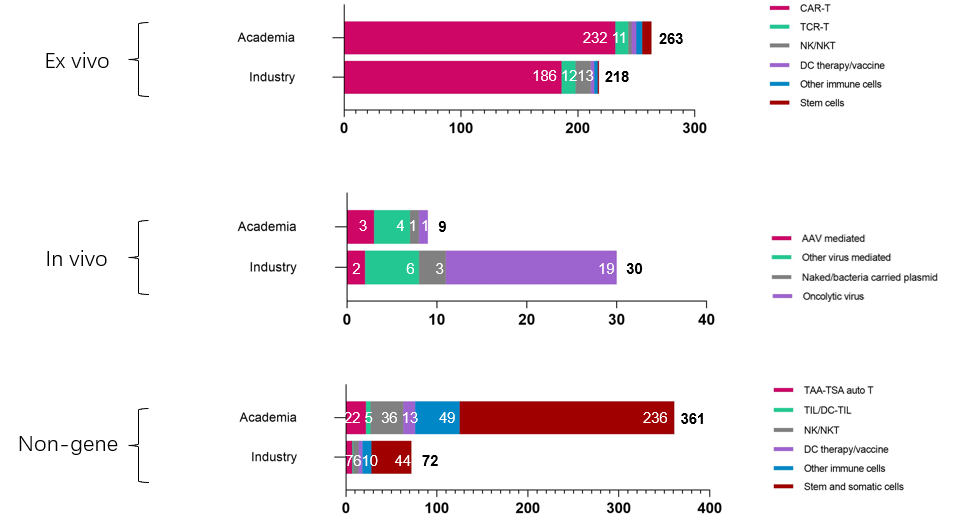

Supplement: Supplementary file 1 — Additional file 1: Supplementary Methods, Table 1–2 and Figure 1. [file 13045_2022_1354_MOESM1_ESM.docx]
